# Alveolar Macrophage Polarisation in Lung Cancer

**DOI:** 10.1155/2014/721087

**Published:** 2014-05-08

**Authors:** Saleh A. Almatroodi, Christine F. McDonald, Dodie S. Pouniotis

**Affiliations:** ^1^Cancer & Tissue Repair Laboratory, RMIT University, Bundoora, VIC 3083, Australia; ^2^Applied Medical Sciences College, Qassim University, Buraidah 51452, Saudi Arabia; ^3^Institute for Breathing & Sleep, Austin Health, Heidelberg, VIC 3084, Australia; ^4^School of Medical Sciences, RMIT University, P.O. Box 71, Bundoora, VIC 3083, Australia

## Abstract

The role of alveolar macrophages in lung cancer is multifaceted and conflicting. Alveolar macrophage secretion of proinflammatory cytokines has been found to enhance antitumour functions, cytostasis (inhibition of tumour growth), and cytotoxicity (macrophage-mediated killing). In contrast, protumour functions of alveolar macrophages in lung cancer have also been indicated. Inhibition of antitumour function via secretion of the anti-inflammatory cytokine IL-10 as well as reduced secretion of proinflammatory cytokines and reduction of mannose receptor expression on alveolar macrophages may contribute to lung cancer progression and metastasis. Alveolar macrophages have also been found to contribute to angiogenesis and tumour growth via the secretion of IL-8 and VEGF. This paper reviews the evidence for a dual role of alveolar macrophages in lung cancer progression.

## 1. Introduction

**Figure 1:**
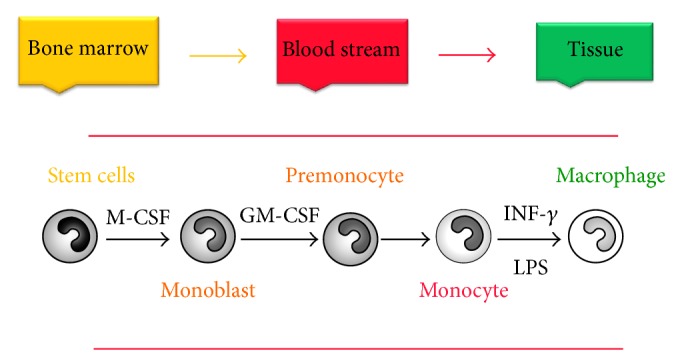
Monocyte and macrophage differentiation. Immature monocytes, released from bone marrow, migrate through the bloodstream under the influence of different cytokines and chemokines into tissues (lung, liver, spleen peritoneal cavity, and brain) where they differentiate into resident macrophages under the influence of IFN-*γ* and LPS.

Alveolar macrophages (AMs) are mononuclear phagocytic cells derived from bone marrow, which migrate as peripheral blood monocytes to the lungs, where they differentiate into AMs through the influence of interferon-*γ* (IFN-*γ*) and lipopolysaccharide (LPS) (Figure 1) [1]. AMs are localised in the airspaces of the lungs [2] and exist in the lower airways in the presence or absence of infection [3], where they play a crucial part in immunity and inflammation of the lungs [1]. Complement receptors are important in the contribution of AMs to initial defence via the production of chemokines such as interleukin-8 (IL-8), monocyte chemoattractant protein-1 (MCP-1), and growth factors. They also function diversely in the regulation of adaptive immunity, including the production of reactive oxygen and nitrogen species (ROS and NOS) and metalloproteinases as well as through antigen presentation [4].

AMs regulate local inflammatory reactions via the release of cytokines and provide a primary defence mechanism against foreign particles by phagocytosis [5]. Cytokines are involved in numerous immune reactions, including tumoricidal activity [6]. AMs secrete both proinflammatory cytokines such as tumour necrosis factor (TNF-*α*), IL-1, IL-6, and IL-12 [7, 8] and anti-inflammatory cytokines such as IL-10, transforming growth factor *β* (TGF-*β*), and IL-13 [9, 10].

Upon stimulation, AMs may exert direct cytotoxic effects by phagocytosis or antitumour effects via cytokine release and natural killer cell activation [11]. AMs therefore appear to play a critical role in lung immunoregulation and potentially in the prevention of lung diseases, including lung cancer [12].

Studies investigating the role of AMs in lung cancer have provided inconsistent results. Whilst some studies report increased cytotoxic activity and antitumour effects after AM activation others have reported decreased cytotoxic activity and protumour effects [6, 7, 9, 11, 13–15]. A dual role for macrophages in lung cancer has therefore been suggested with the idea that AMs may both inhibit and/or promote tumour progression [9].

## 2. Alveolar Macrophages Polarisation and Tumour Progression

**Figure 2:**
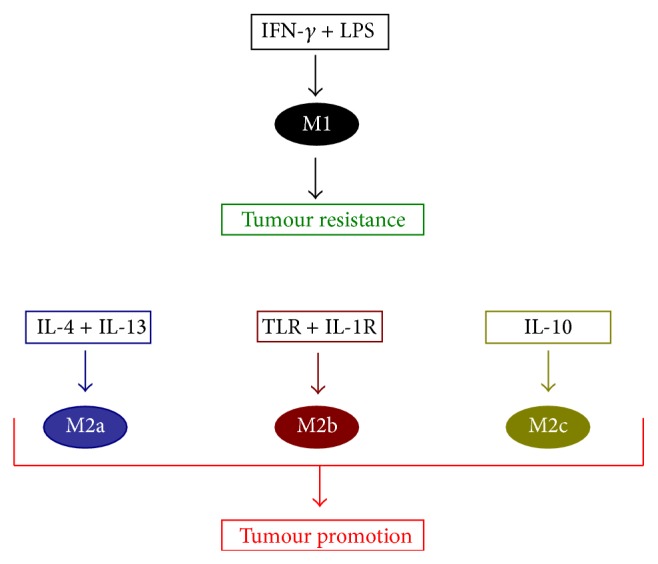
Macrophage subsets have distinctive inducers and multiple functions. Macrophages polarise into M1 subset under the influence of IFN-*γ* and LPS and have antitumour functions via the secretion of effective factors such as iNOS, IL-1, TNF, and IL-6. In contrast, IL-4 and IL-13 stimulate M2a polarisation; M2b is induced by exposure to TLR and IL-1R, and IL-10 is described as an inducer of M2c. The three M2 subtypes are described as tumour promoters; they induce tumour growth through inhibition of efficient factors and/or overexpression of tumour progression factors such as IL-10, TGF-*β*, and angiogenesis mediators.

The role of macrophages in the tumour environment has been widely reviewed. There are two different types of macrophages: classically activated macrophages (M1) and alternatively activated macrophages (M2). The M1 is activated by IFN-*γ* with or without LPS and tumour necrosis factor (TNF-*α*) [16–19]. The M1 macrophage has also been linked to the expression of IL-1, IL-12, TNF-*α*, and inducible nitric oxide synthase (iNOS) [18]. A study has shown the presence of both M1 and M2 phenotypes within nonsmall cell lung cancer (NSCLC) tumour islets [17]. In addition, the M1 macrophage subset has been associated more within tumour islets and with extended survival time in patients with NSCLC (Figure 2) [17].

In contrast, the M2 has three well-defined forms: M2a: induced by IL-4 or IL-13; M2b: induced by exposure to immune complexes and agonists of toll-like receptor (TLRs); and M2c: induced by IL-10 and glucocorticoids (Figure 2) [19].

In addition, macrophages have been shown to exhibit several phenotypes, mainly depending on the environment in which they are found. There are many examples of macrophage-polarising events during tumour progression, including the secretion of tumour-derived mediators and hypoxic tissue damage, as well as influences from other immune cells and stromal components [17, 20–22]. The exact characterisation of AM populations within M1 and M2 subtypes is likely to be overgeneralised, as macrophages have been described as a highly plastic cells that can display a variety of phenotypes [20]. However, markers of M1 and M2 macrophages can still be used to categorise the main phenotype or function of different macrophage populations [20]. A small number of AMs express both M1 and M2 markers and this leads to the suggestion that a mixed phenotype occurs [22]. A study verifies that the M1 and M2 markers differentiate macrophage cell populations although about 5% of the cells were stained for both M1 and M2 markers using immunohistochemistry (IHC) [17].

The M2 macrophage has been associated with tumour initiation and progression and has also been described as an inhibitor of inflammatory responses and adaptive Th1 immunity [16, 17]. M2 macrophages produce anti-inflammatory cytokines such as IL-10 and stimulate the expression of the mannose receptor and arginase I while simultaneously reducing iNOS and arginine [23]. Other than their ability to reduce the amount of NO, which is significant for the elimination of tumour cells [24], M2 can inhibit antigen presentation [19] and T cell proliferation [25]. The cytokines that regulate M2 activation have been associated with NSCLC and other types of tumours [26]. A recent study showed that early lung neoplasia* in vivo* is associated with increased numbers of M2 macrophages regardless of genetic or chemically induced models of carcinogenesis [23]. Also* in vitro*, M2 macrophages have been found to encourage growth of various tumour cells [27] and to increase tumour cell survival [28]. M2 macrophages play a vital role in promoting angiogenesis via production of vascular endothelial growth factor (VEGF), which is a prominent mediator of angiogenesis [17, 29].

## 3. Potential Role of Alveolar Macrophages in Tumour Regression

### 3.1. The Role of Proinflammatory Cytokines: TNF-*α*, IL-1, and lL-6

AMs derived from patients with lung cancer have been shown to function efficiently against tumour growth* in vitro* [6, 7, 11, 13, 30]. Increased TNF-*α* and IL-1 secretion from AMs of patients with lung cancer after stimulation with IFN-*γ* and granulocyte-macrophage colony-stimulating factor (GM-CSF) has been demonstrated in comparison with patients with nonmalignant disorders [7]. IL-1 mediates cytotoxicity and suppression of tumour growth [31, 32] and TNF-*α* inhibits angiogenesis to prevent tumour growth. TNF-*α* can therefore act as an antitumour monokine and be responsible for spontaneous (without external activation) cytotoxic effects of AMs [33]. Hence, AMs from patients with lung cancer may be able to secrete normal amounts of proinflammatory cytokines, suggesting that AMs function normally in patients with lung cancer. Despite these findings, decreased TNF-*α* secretion by AMs has also been reported in lung cancer patients [9, 15, 32]. However, Lentsch et al. showed that the reduction in TNF-*α* did not impair cytotoxic activity of AMs against lung cancer* in vivo* [32]. Overall, secretion of TNF-*α* by AMs appears to have an important antitumour role; however there may be other mechanisms that contribute to their antitumour effects in the absence of TNF-*α*.

Numerous studies have isolated AMs from bronchoalveolar lavage (BAL) for assessment of their function in the presence of known macrophage-activating agents such as IFN-*γ* and GM-CSF [8, 13, 15, 31, 34]. IFN-*γ* stimulates IFN-*γ* inducible protein (IP-10), an antitumour molecule that impairs tumour angiogenesis, whereas GM-CSF promotes proliferation of haematopoietic progenitor cells and influences the antitumour function of AMs [34]. After IFN-*γ* stimulation, the cytotoxic effects of AMs in lung cancer patients are enhanced even from a depressed baseline level [11, 31]. Stimulation with GM-CSF enhanced expression of mRNA coding of TNF-*α*, IL-1, and IL-6 in AMs and monocytes from patients with lung cancer in a time-dependent manner and isolation of AMs from patients with lung cancer receiving GM-CSF therapy and cultured with LPS showed enhanced IL-6 secretion [8, 34]. These findings suggest that GM-CSF therapy may enhance tumoricidal activity of AMs in patients with lung cancer.

The ability of IL-6 to inhibit tumour cell growth has been suggested [6]. Significantly elevated IL-6 levels have been detected in BAL cell cultures and blood from patients with lung cancer compared to those with benign disease [6]. Upon LPS stimulation, IL-6 secretion was increased by AMs from patients with lung cancer [6]. IL-6 functions synchronously with IL-1 and TNF-*α* in order to support antitumour immunity [34]. A recent study investigated IL-6 levels in BAL fluid and serum from patients with lung cancer before and during radiotherapy (RT). IL-6 levels in BAL fluid were higher compared to controls and were further elevated during RT, potentially confirming the role of IL-6 in mediating an inflammatory response. However, the study did not specify the source of IL-6 secretion [35].

However, other studies have demonstrated the ability of IL-6 to promote lung tumour growth and its association with poor prognosis [36–38]. A study has shown that tumour growth caused by tobacco smoke was mediated by IL-6 and TNF-*α* [36]. Also a deficiency of IL-6 and TNF-*α* leads to decreased lung tumour cell proliferation [36]. Similarly, another study has shown that IL-6 plays a vital role in promoting cancer stem cells derived from a NSCLC cell line H460 [37]. It was suggested that targeting IL-6 could potentially improve lung cancer therapeutic techniques [37]. An interesting clinical study demonstrated that NSCLC patients with high plasma levels of IL-6 responded poorly to chemotherapy. Also they suggested that IL-6 could be used as a prognostic marker for lung cancer survival among those patients who have received chemotherapy [39].

## 4. Potential Role of Alveolar Macrophages in Tumour Progression

### 4.1. The Role of Anti-Inflammatory Cytokines: TGF-*β* and IL-10

AMs in lung cancer have been shown to exert protumour effects via their secretion of anti-inflammatory/immunosuppressive cytokines such as TGF-*β* and IL-10 [9, 13]. TGF-*β* has been shown to enhance tumour cell proliferation* in vitro* and* in vivo* [9].* In vitro* stimulation of AMs with LPS induced TGF-*β* secretion in patients with lung cancer [6]. Also, increased TGF-*β* secretion has also been reported in the serum of lung cancer patients compared to healthy controls [40]. However, the mechanism underlying the increased production of TGF-*β* and its role on AM regulation and lung cancer growth is unclear [40].

IL-10 is a potent tumour angiogenesis inhibitor released by AMs with or without LPS stimulation* in vitro*. Following LPS stimulation, Kataki et al. found no significant differences in IL-10 production by AMs from patients with lung cancer in comparison with control patients [9, 13]. Conversely, IL-10 has been identified as a potential inhibitor of proinflammatory cytokines and other cytotoxic molecules that mediates the killing of tumour cells. IL-10 is therefore found to inhibit the antitumour activities of AMs and may contribute to tumour progression [41].

### 4.2. Altered Alveolar Macrophages Function via Inhibition of Antitumour Effects

AMs have also been shown to function inefficiently and to promote tumour growth in patients with lung cancer [11, 14, 15, 32, 41]. Inhibition of proinflammatory cytokine secretion by AMs has been reported in the presence of elevated levels of serum IL-10 [32, 41]. In murine peritoneal macrophages, LPS-induced TNF-*α* release was inhibited by IL-10 [42]. The suppression of TNF-*α* function favours tumour proliferation and differentiation through the activation of IL-10. This is consistent with the suppression of AM antitumour function in advanced stage lung cancer [12].

IL-10 secretion by AMs has also been found to inhibit other T cell proliferation inducers, such as IL-2 [32]. Although the antitumour effect of IL-2 is not completely understood, it has the capacity to mediate cytotoxicity and synthesise IL-1 and TNF-*α* [43]. In addition, IL-10 decreased golgi apparatus (GA) and rough endoplasmic reticulum (RER) function in AMs from patients with lung cancer [32]. Since the GA and RER allow for the translation of mRNA into protein and thus synthesis and secretion of cytokines, such as TNF-*α*, dysfunction in these organelles is likely to downregulate AM function. A reduction in TNF-*α* could lead to a reduction in cytostatic activity of AMs and may promote tumour growth and proliferation [32].

Cytostatic activity of AMs is compromised in patients with lung cancer [11, 44–46]. In some studies, the reduction in cytostatic activity is greater in AMs from tumour-bearing segments compared to non-tumour-bearing segments [45], whereas in others the defect appears more generalised [11].

### 4.3. Altered Cytotoxicity and Cytokine Release

Altered cytotoxic activity of AMs from patients with lung cancer has been demonstrated [7, 9, 14, 15] with both increased [7, 47] and decreased [9, 15] cytotoxic function described. Impaired cytotoxic ability of AMs from patients with lung cancer has been shown along with increased TNF-*α* and IL-1 production in the same patients [7]. This revealed an obvious discrepancy in the correlation between cytokine release and cytotoxicity. Increased cytotoxic activity by AMs from lung cancer patients was discovered, however, with no reported alteration in cytokine release [47]. These results suggest that increased cytokine release or cytotoxicity may be a reaction to the presence of tumour, rather than a primary mechanism preventing its occurrence. However, altered cytotoxic activity in AMs and tumour-associated macrophages (TAMs) has been shown to be mediated via decreased secretion of cytokines (IL-1, IL-6, and TNF-*α*) in patients with lung cancer [9, 15]. Decreased TNF-*α* and IL-1 secretion has been demonstrated in AMs from patients with both NSCLC (squamous and large cell undifferentiated subtypes) and small cell lung cancer and reduced IL-6 secretion has been demonstrated from AMs derived from patients with large cell undifferentiated and small cell subtypes [15]. TNF-*α*, IL-1, and IL-6 promote the induction of Th1 cells, which enable macrophage-mediated killing. Reduced Th1 mediated cytokines may consequently limit the cytotoxic potential of AMs or TAMs and enable tumour progression [9].

Whilst IL-6 release can contribute to the antitumour function of AMs, a protumour function has also been proposed. Even with IFN-*γ* and LPS stimulation, IL-6 inhibited the development of tumoricidal function in AMs of patients with lung cancer [14]. Overproduction or dysregulation of IL-6 by macrophages can contribute to the growth and metastasis of many carcinomas, including lung cancer [48].

### 4.4. Altered Phagocytic Capacity and Receptor Expression

Decreased antibody-mediated cytotoxicity in AMs from lung cancer patients has been reported. AMs normally take up antigens for processing and present them on major histocompatibility complex (MHC) classes I and II molecules. This activates T cells and in turn stimulates phagocytosis (macrophage-mediated killing) and the release of proinflammatory cytokines (TNF*α* and IL-1) [5]. Pouniotis et al. (2006) found that AMs from patients with small cell and squamous cell carcinoma were impaired in their ability to uptake 40 nm fluorescent polystyrene beads, in comparison with controls, while AMs from patients with small cell, squamous, and large undifferentiated carcinoma showed impaired uptake of 1000 nm beads [15]. Impairment of uptake ability may highlight impairment in the phagocytic ability of AMs in lung cancer. This study suggested differences in the phagocytic ability of AMs depending upon histological subtype of lung cancer. Reduced functionality of AMs may promote tumour growth and differentiation.

Altered cell surface expression of MHC molecules on AMs has been found in patients with lung cancer. Decreased MHC class II expression on AMs from patients with small cell carcinoma and decreased intracellular adhesion molecule (ICAM-1) and CD83 expression from patients with small, large, and squamous cell carcinoma after IFN-*γ* stimulation have been demonstrated by flow cytometry [15]. Conversely, McDonald and Atkins found no difference in the expression of ICAM-1 with or without IFN-*γ* stimulation [11]. ICAM-1 plays an important role in the early stages of T cell activation. Reduced expression of ICAM-1 therefore impairs AM-mediated cytotoxicity [5]. In addition, reduced expression of mannose receptor by AMs in lung cancer patients has also been shown [15]. Mannose receptor is a transmembrane glycoprotein that binds and internalises carbohydrate ligands such as mannose and fructose. Mannose receptor is expressed on AMs and plays a role in host defence and immune regulation. Therefore reduced receptor expression may impair antitumour function by AMs and favour tumour progression [49].

Alterations in the functional status of AMs vary with the stage of lung cancer [12, 45, 47]. A decline in cytostatic and cytotoxic activity of AMs from patients with advanced stages of primary lung cancer has been observed [12, 45, 46] suggesting a growth-promoting effect of lung tumours on AM function. In contrast, increased cytotoxicity in advanced stages of lung cancer has also been documented [47].

The underlying demographic and clinical characteristics of a patient may also influence AM functions. Such characteristics include age, smoking status, tumour histology, tumour stage, and the presence of underlying comorbidities such as chronic obstructive pulmonary disease (COPD). BAL and whole blood cells from patients with small cell lung carcinoma and NSCLC cancer were compared in their secretion of IL-1, IL-6, and TNF-*α*. They suggested that BAL and blood cells from patients with small cell lung carcinoma secreted significantly less cytokines (IL-1, IL-6 and TNF-*α*) than BAL and blood cells from patients with NSCLC [6]. Although Pouniotis et al. noted differences in function between AMs from patients with different histological subtypes of lung cancer [15], the majority of studies do not differentiate further than comparing between the two major categories of small cell lung carcinoma or NSCLC [7, 9, 13, 50].

## 5. Role of Alveolar Macrophages in Angiogenesis

AMs can promote tumour progression by facilitating angiogenesis. Angiogenesis plays an important role in tumour growth, invasion, and metastasis [48]. It involves degradation of extracellular matrix and invasion of stroma by endothelial cells [48]. The proliferation, migration, and differentiation of endothelial cells into functional capillaries initiate the development of vasculature in the tumour [5]. AMs in lung cancer release growth factors, cytokines, and inflammatory mediators, which regulate angiogenesis. These include IL-8, VEGF, platelet derived growth factor (PDGF), basic fibroblast growth factor (bFGF), and matrix metalloproteinases (MMPs) [51].

Increased secretion of IL-8 by AMs has been reported in patients with lung cancer and increased gene expression of IL-8 in lung cancer cell lines after coculture with TAMs has also been found [51]. Since IL-8 is a potent angiogenic factor released from AMs, increased gene expression and cytokine secretion may favour tumour growth and metastasis [34]. IL-8 in serum and BAL fluid has been shown to be higher in patients with lung cancer than in noncancer controls [35] and the level of IL-8 in BAL fluid can be considered as a prognostic factor for decreased survival [35]. Lung cancer cells from patients with NSCLC demonstrated increased IL-8 mRNA expression after interaction with tumour infiltrating macrophages* in vitro*, suggesting that tumour cells may be activated by macrophages and secrete angiogenic factors (such as IL-8) which enable tumour progression [51]. Tumour progression associated with reduced survival rates has also been found in patients with NSCLC whose tumour cell lines demonstrated increased IL-8 mRNA expression compared to those with reduced expression [51].

VEGF also plays an important role in angiogenesis [29]. Increased transcription and protein production of VEGF have been reported in multinuclear giant cells of pulmonary sarcoidal granulomas [52]. VEGF may have a chemotactic effect on TAMs and increased expression may guide migration to avascular areas, increasing blood vessel development in tumour cells [5]. The expression of VEGF-C, a member of the VEGF family, has been shown in tumour cells and stromal macrophages of patients with NSCLC using IHC technique. However, the VEGF-C status in tumour cells and stromal macrophages did not correlate with nodal metastasis or angiogenesis [53]. The underlying mechanisms involved in VEGF expression in AMs and their functional significance in lung cancer remain incompletely determined [54].

Increased PDGF production by TAMs in tumour stroma has been reported [9, 50]. Tumour stroma is specialised peritumoral connective tissue comprised of endothelial, mesenchymal, and inflammatory cells [9]. It is suggested that elevated PDGF in tumour stroma favours tumour progression and angiogenesis via the migration of endothelial and mesenchymal cells [9]. This is supported by studies showing that PDGF stimulates cell migration in human lung carcinoma cells [55]. Cell migration plays an important role in metastasis and can be promoted via the release of cytokines from AMs.

MMPs are matrix degrading enzymes that facilitate tumour growth through the breakdown of basement membranes [5]. In patients with lung cancer, macrophage elastase (MMP-12) and gelatinase (MMP-9) have been shown to promote lung tumour growth [56, 57]. MMP-12 is released by AMs and favours tumour progression by enhancing angiogenesis and the breakdown of elastin. Interestingly, an association between increased VEGF and MMP-12 gene expression and tumour vascularity has been demonstrated [56]. MMPs are an attractive target for therapeutic purposes because of their involvement in tumour progression. However, a recent trial has suggested that some MMPs might play a crucial role in host resistance against tumour progression. An increase in Lewis lung carcinoma (LLC) pulmonary metastasis was shown in MMP12-deficient mice, while regular MMP12 expression was associated with reduced tumour-associated microvessel density* in vivo* [58]. Collectively, these results suggest an important role for MMP12 in inhibiting lung metastasis and indicate that MMP inhibitors could be designed to target tumour-promoting MMPs in order to inhibit tumour growth [58].

Increased MMP-9 levels and significant correlations between tumour stage and both BAL fluid and plasma MMP9 levels have been demonstrated in patients with NSCLC [59]. It has also been suggested that serum MMP-9 levels (but not BAL fluid MMP-9 levels) can be useful to distinguish between malignant and benign lung diseases. The same authors also demonstrated that serum MMP-9 levels relate to both disease stage and general clinical status of patients with NSCLC [60]. These studies did not, however, specify which cells (macrophages or cancer cells) were secreting the MMP-9. Further investigation is clearly required into the relationship between AMs, MMPs, and lung cancer.

## 6. Altered Alveolar Macrophages Function: The Role of Smoking

Smoking increases the risk of lung cancer. A number of studies have examined the impact of smoking on AM functionality [47, 54, 61–64]. Some have shown suppressed antitumour activity of AMs from smokers with lung cancer compared to current nonsmokers with the disease [12]. Using LPS and IFN-*γ* stimulation, cytotoxicity levels were reduced in AMs from current smoking patients with lung cancer, compared to nonsmokers and exsmokers. Cigarette smoke contains approximately 4000 components, 55 of which are described as carcinogenic. One of the most important nicotine derivatives is the nitrosamine 4 (methylnitrosamino)-1-(3-pyridyl)-1-butanone (NNK). NNK has been found to downregulate cytokine production by AMs. NNK activation inhibits TNF and IL-12 while increasing IL-10 and PGD2 release. These results suggest that NNK favours the differentiation of AMs from the M1 to the M2 phenotype, which is known to induce tumour growth, angiogenesis, and progression [62].

The impact of cigarette smoking on AM recognition molecules and phagocytic capacity in a noncancer context was investigated in another study which found decreased AM phagocytic ability in both current smokers and exsmokers with COPD and healthy smokers when compared to nonsmoking control subjects. However, phagocytic ability was greater in patients COPD who had ceased smoking compared to current smokers with the condition. It has also been demonstrated that smoking impacts AM recognition molecules and is associated with cyclic adenosine monophosphate (cAMP) increase [63].

A recent study showed that cigarette smoke is able to induce AM polarisation towards M2 phenotype in patients with COPD by deactivating M1. Researchers found that cigarette smoke was able to downregulate M1-related genes such as CXCL9, CXCL10, CXCL11, and CCL5, while upregulating M2-related genes such as MMP-2 and MMP-7 [65]. Further investigation is required to determine the polarisation status of AMs within the tumour microenvironment of patients with lung cancer, which factors favour AM polarisation, and whether alterations in polarisation modulate tumour growth and prognosis.

Downregulation of IFN-*γ* signalling has been demonstrated in AMs from smokers. IFN-*γ* is an important cytokine, which can perform several functions important for the development of activated macrophages during inflammation. Dhillon and colleagues found that chronic exposure of the alveolar space to cigarette smoke could result in a loss of IFN-*γ*R *α*-chain expression on AMs, followed by a downregulation of IFN-*γ* signalling [64]. The function of IFN-*γ* in immune surveillance may thus be deficient in the lungs of smokers and could lead to both infection and the development of cancer [64]. Many of the studies examining smoking and its effect on AMs have utilised different animal models of smoking. Although the translation from animal to human studies is still not clear, animal studies are able to provide greater focus on the interaction between cigarette smoking and AM function. One study has shown that the release of MMP-12 and TNF-*α* by AMs was altered in mice exposed to cigarette smoke. MMP-12 secretion from mouse AMs was increased after cigarette smoke exposure, whereas TNF-*α* secretion showed an initial time-dependent increase and subsequent decrease over ten days [61]. Cigarette smoke may thus progressively compromise AM functionality, particularly in terms of TNF-*α* secretion. However, more research is required to investigate the role of cigarette smoking on proinflammatory and MMP secretion from AMs and its association with lung cancer.

## 7. Alveolar Macrophages: Roles of Reactive Nitrogen/Oxygen Species (ROS and NOS)

AMs release large amounts of toxic molecules such as ROS and NOS [66]. Overproduction of ROS/NOS has been described as a potential factor leading to cancer through mechanisms such as inhibition of apoptosis, DNA damage, and P53 mutation [67]. AMs taken from tumour-bearing lobes of patients with lung cancer were demonstrated in a study by Sharma et al. to produce significantly higher quantities of oxygen radicals compared with those from disease-free lobes [68].

AMs are one of the main producers of cytokines and their production may be modified in response to alterations in NOS concentration. In studies which have investigated the role of NOS in human lung cancer [69–71], increasing NOS levels in BAL fluid were associated with squamous cell lung carcinoma [70]. Additionally, the generation of NOS was higher in cultured AMs from patients with lung cancer in comparison to controls [71]. NOS is able to regulate VEGF expression in mouse lung tumour as well as other systems, demonstrating NOS ability to modulate angiogenesis in lung tumours* in vivo*. Kisley et al. (2002) suggested that the absence of iNOS or inhibitors of its activity reduced mouse lung cancer development [72]. The same results have also been achieved in studies of rodent cancer models [73]. Most of the studies showing a clear role of macrophage-mediated NOS secretion in tumour inhibition have been in murine models and its role in human macrophages requires further investigation [74].

## 8. Conclusion

The role of AMs in lung cancer appears paradoxical. Whilst clearly implicated in inhibiting tumour growth with consequent tumour regression, AMs have also been demonstrated to have protumour functions resulting in tumour progression. Differences between studies may relate to the examination of different lung cancer histological subtypes, different tumour stages, or examination of AMs from different lung segments (i.e., tumour-bearing or non-tumour-bearing lung segments). Patient demographics such as smoking status and the presence or absence of comorbidities such as COPD may also contribute to differences. A number of pro- and/or anti-inflammatory cytokines have been described as possessing dual roles in lung cancer including TNF*α*, IL-6, IL-8, IL-10, VEGF, and PDGF. However, their direct effect on the function of AMs in patients with lung cancer is not completely understood. Smoking has also been associated with altered macrophage functionality in lung cancer patients. Future studies are encouraged to examine AM function in lung cancer under a range of different conditions including diverse histological subtypes and to relate this to patient response to treatment and prognosis.

## Abbreviations

AMs: Alveolar macrophages
LPS: Lipopolysaccharide
IFN-*γ*: Interferon-*γ*
IL-: Interleukin
MCP-1: Monocyte chemoattractant protein-1
TNF: Tumour necrosis factor
TGF: Transforming growth factor
iNOS: Inducible nitric oxide synthase
M1: Classically activated macrophages
M2: Alternatively activated macrophages
NSCLC: Nonsmall cell lung carcinoma
VEGF: Vascular endothelial growth factor
GM-CSF: Granulocyte-macrophage colony-stimulating factor
MMPs: Matrix metalloproteinases
IHC: Immunohistochemistry
MHC: Histocompatibility complex.
